# Supplementary material for: Biologic Brachytherapy: Genetically Modified Surgical Flap as a Therapeutic Tool—A Systematic Review of Animal Studies
Source: Int J Mol Sci. 2024 Sep 25;25(19):10330. doi: 10.3390/ijms251910330 (PMC11476562; doi:10.3390/ijms251910330)
Supplement: Supplementary file 1 [file ijms-25-10330-s001.zip › ijms-3213319-supplementary.pdf]

File S1: Full study protocol

Searches

The following electronic bibliographic databases have been searched from first record to the present: PubMed, EMBASE, Scopus and Web of Science. A Boolean search strategy was used to combine keywords and subject headings for flap with those for genetic modification. No language restrictions were imposed. All references were imported into EndNote and duplicates deleted. We will also carry out bibliographic screening and citation searching (using the Web of Science citation search tool) of the included papers and any existing reviews identified as being of relevance, as well as consulting experts.

Search strategy

https://www.crd.york.ac.uk/PROSPEROFILES/359982_STRATEGY_20220913.pdf

Study designs to be included

Inclusion criteria: We will include studies that compare at least one control group with at least one treatment group.

Exclusion criteria: 1) Solely in vitro study

Human disease modelled Surgical flap necrosis/ischemia

Animals/population

Inclusion criteria: All animal models of surgical flaps included. No restrictions imposed.

Exclusion criteria: 1) Human studies 2) Split-thickness skin grafts

Intervention(s), exposure(s)

Inclusion criteria: Genetic modification of a tissue bulk of a living organism or genetic modification of cells/tissues later integrated with the former tissue bulk, Use of the above-mentioned tissue as a surgical flap

Exclusion criteria: 1) Additional ischemia after the intervention

Comparator(s)/control

Inclusion criteria: Sham genetic modification of a tissue bulk or treatment using established standard

Exclusion criteria: 1) Lack of intervention in the control group

Other selection criteria or limitations applied

Exclusion criteria: 1) Not relevant publication type (conference abstracts, reviews, reviews editorials, and other non-primary studies) 2) Severe flaws/missed data in reporting 3) Studies reported in languages other than English

Study selection and data extraction

Titles and abstracts of studies retrieved using the search strategy and those from additional sources will be screened using Rayyan QCRI to identify studies that potentially meet the inclusion criteria outlined above. The full text of these papers will be retrieved and screened to confirm inclusion status, and if excluded at this stage reason for exclusion will be noted. All screening will be carried out

independently by two review team members. Any disagreement between them over the eligibility of particular studies will be resolved through discussion, with a third author where necessary

Methods for data extraction

A standardised, pre-piloted Excel spreadsheet will be used to extract data from the included studies. Two review authors will extract data independently, and discrepancies will be identified and resolved through discussion, with a third author where necessary. Missing data will be requested from study authors where appropriate via email
